# Supplementary material for: Evaluation of choroidal thickness in prodromal Alzheimer’s disease defined by amyloid PET
Source: PLoS One. 2020 Sep 21;15(9):e0239484. doi: 10.1371/journal.pone.0239484 (PMC7505462; doi:10.1371/journal.pone.0239484)
Supplement: Attachment — Submitted filename: corrections2.docx [file pone.0239484.s001.docx]

PONE-D-20-16899R1
Evaluation of choroidal thickness in prodromal Alzheimer´s disease defined by amyloid PET
Mrs Alicia López de Eguileta

Dear Dr. López de Eguileta,

We've checked your submission and before we can proceed, we need you to address the following issues:

1..Thank you for responding to our previous regarding your manuscript. We note that the participants included patients with dementia. Please clarify how patients with dementia were determined capable of providing informed consent. If you did not assess the capacity to provide consent please outline why this was not necessary in this case. Thank you for your attention to this request.

As you suggested, to clarified how patients with dementia were determined capable of providing informed consent, we added this in Methods section in the manuscript: ”All patients enrolled were able to understand the information contained in the written consent and they were not legally incompetent.“

2..Based on the information you have provided, we propose the following funding and competing interests for your approval:
Funding:
Dr. Sánchez-Juan was supported by grants from IDIVAL, Instituto de Salud Carlos III (Fondo de Investigación Sanitario, PI08/0139, PI12/02288, PI16/01652, JPND (DEMTEST PI11/03028) and the CIBERNED program and Siemens Healthineers (Valdecilla Cohort for Memory and Brain Aging). The funders had no role in study design, data collection and analysis, decision to publish, or preparation of the manuscript.

Competing interests:
Dr. Sánchez-Juan received funding from Siemens Healthineers. This does not alter our adherence to PLOS ONE policies on sharing data and materials.

Can you please confirm in your cover letter the above statements are both complete and correct? With your approval we will update your competing interests and funding statements on your behalf.

We confirmed the above statements are both complete and correct and we added them in the Cover letter.
